# Effect of circulating prolactin, lactation days, and seasonal variations on first artificial insemination pregnancy rates using the PG7G protocol in dairy cows

**DOI:** 10.14202/vetworld.2025.40-51

**Published:** 2025-01-09

**Authors:** Mufeed A. Alnimer, Mohamed A. Abedal-Majed, Mohmmad Al-Qaisi, Ahmad I. Shamoun

**Affiliations:** Department of Animal Production, School of Agriculture, The University of Jordan, Amman, 11942, Jordan Amman

**Keywords:** dairy cows, days in milk, pregnancy rate, prolactin, season, timed artificial insemination

## Abstract

**Background and Aim:**

Dairy farm profitability is linked to milk yield and reproductive efficiency. High prolactin levels during lactation can negatively impact fertility. Timed AI protocols like PG7G are used to improve pregnancy rates. This study investigates the effects of extending the voluntary waiting period (VWP) from 63 to 73 days after the PG7G protocol on reproductive parameters, including progesterone and prolactin levels, pregnancy rates, and pregnancy losses, in lactating Holstein cows during summer and winter seasons.

**Materials and Methods:**

A total of 2100 lactating dairy cows were divided into groups and assigned to the PG7G protocol based on their parity. Two groups were formed based on the number of days in milk (DIM) after the first PGF_2α_ injection. The first group (PG7G-63) received a PGF_2α_ injection 30-day postpartum (pp) and followed a specific protocol. The second group, PG7G-73, followed the same protocol but received a PGF_2α_ injection 40-day pp. Pregnancy was confirmed, and blood samples were collected for analysis. Temperature and humidity were also recorded throughout the study.

**Results:**

Extension of the VWP to 73-day (PG7G-73 group) significantly improved pregnancy rates on day 47 pp compared with the 63-day VWP (PG7G-63 group). However, the PG7G-63 group exhibited lower PLs. Seasonality markedly influenced reproductive outcomes, with higher P/AI in the moderate season for primiparous cows and in the hot season for multiparous cows. Conversely, PL in both groups increased during the hot season.

**Conclusion:**

Extending the VWP from 63 to 73 days pp in lactating Holstein cows significantly improved pregnancy rates, while seasonality affected reproductive outcomes, with higher pregnancy rates in moderate temperatures and increased pregnancy loss during hot seasons.

## INTRODUCTION

Dairy farms’ profitability is significantly influenced by milk yield and reproductive efficiency [1, 2]. The increasing selection pressure for higher milk production has negatively affected dairy cow fertility [3, 4]. Prolactin (PRL), a hormone linked to milk production, is associated with impaired ovarian function [5]. Elevated PRL levels during lactation can inhibit gonadotropin secretion, leading to anovulation and infertility [6, 7]. Previously, blood serum PRL levels were observed to increase before parturition and then decline during the first 6 weeks postpartum (pp) [8]. The role of PRL in regulating ruminant lactation is somewhat unclear, as PRL appears to be modulated at the mammary gland level. Postpartum ovarian inactivity and difficulties in detecting estrus are major reproductive challenges in high-producing dairy cows [9, 10]. Timed artificial insemination (TAI) has been used to overcome the inefficiency of estrus detection [11, 12]. TAI protocols, such as Ovsynch and its modifications (G6G, G7G), have been developed to improve pregnancy rates [11, 13–15]. While these protocols have increased pregnancy per artificial insemination (P/AI) rates, factors such as body condition score (BCS) [16], stage of the estrous cycle at the initiation of the protocol [17], parity [18, 19], and heat stress [20] can influence their effectiveness. Dirandeh *et al*. [21] and Shahzad *et al*. [22] investigated the effects of a prostaglandin F_2α_ (PGF_2α_) treatment administered 14 days before initiating the G6G or G7G synchronization protocol on ovarian response, plasma progesterone (P4) concentration, P/AI, and pregnancy loss (PL) in multiparous Holstein cows. This study found that cows receiving the PGF_2α_ injection exhibited an increased ovulatory response to the first GnRH of Ovsynch, higher P4 concentrations, and improved P/AI on day 60 compared with cows in the G6G and G7G control groups. Collectively, these modifications to the Ovsynch protocol have resulted in P/AI at the first service surpassing 50% in high-producing dairy cows [16, 23], helping dairy farmers to employ more effective reproductive strategies [24, 25].

A longer interval between calving and conception (days open) negatively impacts dairy farm profitability [1]. To maximize returns, dairy farmers must minimize the number of days that their cows stay open. Pp anovulation, occurring in 6%–59% of high-producing dairy cows, reduces pregnancy rates and increases PL [26]. These cows had a lower pregnancy rate per AI and more PLs.

Our previous research by Alnimer *et al*. [19] and Alnimer *et al*. [27] demonstrated the effectiveness of the Ovsynch, Presynch Ovsynch, and Modified Ovsynch protocols in synchronizing cows for timed AI (TAI) between 52 and 55 days in milk (DIM). In a different study, El-Tarabany [28] reported an optimal conception rate achieved between 51–65 DIM. Moreover, Dirandeh [29] achieved higher pregnancy rates by initiating Ovsynch on day 6 of the first pp estrous cycle (40-day pp). However, other studies by Astiz and Fargas [30], Yousuf *et al*. [31], and Heidari *et al*. [32] reported varying numbers of DIM at first service for cows synchronized with the G6G protocol (87.4 ±10.9 days, 76–82 days, and 50 ± 3 days, respectively). Building upon previous research by Dirandeh *et al*. [21], who examined the effects of PGF_2α_ treatment on reproductive parameters in multiparous Holstein cows, our study adopted a different approach. While the earlier investigation focused on administering PGF_2α_ 14 days before G6G and G7G synchronization protocol treatment in multiparous Holstein cows, our objective was to explore the impact of extending the duration of the voluntary waiting period (VWP) from 63 to 73 DIM after the PG7G synchronization protocol on progesterone concentration, PRL concentration, P/AI, and PL in lactating Holstein cows during the summer and winter seasons.

## MATERIALS AND METHODS

### Ethical approval

This study was approved by the Animal Care and Use Committee, University of Jordan (Approval no. 2162).

### Study period and location

The study was conducted from January 2020 to December 2021 at a commercial dairy farm (Alkhaldia area, Al-Mafraq Governate, Jordan) at 32^o^2’ N, 35^o^51’ E.

### Cows, housing, and management

Holstein–Friesian lactating dairy cows were housed in naturally ventilated free-stall barns equipped with shade and sprinkler systems. The sprinkler nozzles were positioned 2 m apart on the pipes, providing a 180° spray toward the back of the cows and bedded with sand. The herd size was approximately 2500 lactating cows with 1300 milking cows. The cows were milked three times daily at 8 h intervals, producing an average milk yield of 9500–10,000 kg per cow per lactation. Milk production was recorded daily for all cows from calving to 120-day pp. Cows were fed a total mixed ration (TMR) consisting of 40% forage (corn silage and alfalfa hay) and 60% concentrate (corn, barley, wheat bran, soybean meal, and commercial concentrate for lactation with trace minerals and vitamins). The TMR provided 1.8 Mcal net energy of lactation (NE_L_)/kg and 19% crude protein (CP) on a dry matter (DM) basis, formulated according to the National Research Council [33] with BCS ranging from 2.75 to 3.25 (1 = emaciated to 5 = obese). The cows had free access to fresh water. Meteorological data such as daily maximum and minimum temperatures and relative humidity (RH) were collected using EasyLog Data loggers (Lascar Electronics, UK). Data loggers were positioned at a height of approximately 2 m within a specialized open-side box enclosure. Throughout the experimental period, the data logger recorded the ambient temperature and RH every hour. This information was used to calculate the temperature humidity index (THI) for each day using the following equation: THI = (1.8 × T °C + 32) − (0.55 − 0.0055 × RH%) × (1.8 × T °C − 26) [34]. The mean maximum temperature (35.6 ± 0.2°C and 22.0 ± 0.1°C), minimum temperature (18.1 ± 0.2°C and 8.9 ± 0.1°C), and THI (81.3 ± 0.3 and 66.8 ± 0.2) were recorded during the experimental period for hot (June–September) and moderate (October–May) months, respectively.

### Experimental design

**Figure 1:**
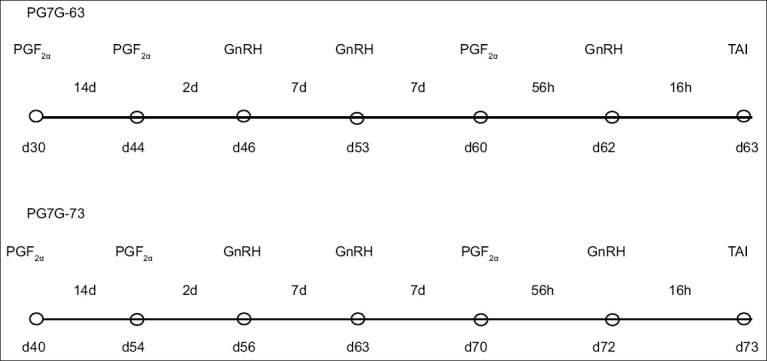
A diagram showing the different hormonal treatment groups of the lactating cows in the study.

A total of 2198 lactating Holstein Friesian dairy cows were initially enrolled. After excluding 98 cows due to clinical mastitis, lameness, and culling, 2100 cows were stratified by parity (primiparous, n = 781 and multiparous, n = 1319) and assigned to the PG7G protocol [21]. Cows were randomly assigned into two groups based on DIM at the first PGF_2α_ injection:

(1) PG7G-63 group (n = 1034): Cows received the PGF_2α_ injection (500 μg cloprostenol, i. m.; 2 mL Estrumate Intervet) on day 30 pp, followed by another PGF_2α_ injection on day 44 pp. Two days later, each cow received an injection of 10 μg GnRH agonist on day 46 pp (Buserelin, Receptal®, Hoechst Roussel Vet GmbH). A modified Ovsynch protocol (Ovsynch56) [35] was initiated on day 53 with GnRH, followed by PGF_2α_ on day 60, a second GnRH on day 62, and TAI on day 63 pp; (2) PG7G-73 group (n = 1066): Cows followed the same PG7G-63 protocol but initiated the first PGF_2α_ injection on day 40 pp, with TAI on day 73 pp (Figure 1). Two skilled AI technicians performed insemination using commercially available frozen semen of proven fertility (WWSires, Avenida de los Robles Visalia, CA, USA). The semen source was randomized among the groups. In addition, routine semen was examined every 2 months to ensure that the semen quality did not change. Pregnancy was diagnosed by ultrasound (Scanner 100 Vet, Pie Medical, Maastricht, The Netherlands) on day 33 ± 3 post-insemination using a 7.5-MHz probe. Pregnancy was confirmed by visualization of an embryonic vesicle with a heartbeat, as previously described by Pierson and Ginther [36]. Pregnancy status was re-evaluated through rectal palpation on day 47 ± 3 post-insemination; PL was calculated as the difference between pregnancy rates at the first and second examinations.

### Blood sample collection and analysis

Blood samples (4.5 mL) were collected through coccygeal venipuncture from 20 cows (10 per group) into heparinized tubes at calving on days 53, 60, and 63 in the PG7G-63 group and on days 63, 70, and 73 in the PG7G-73 group. In addition, samples were collected 7 and 14-day post-TAI in both groups. Samples were centrifuged (1000× *g* for 15 min.) and plasma was harvested and stored at −20°C until assayed for progesterone (P4) and PRL concentrations using an ELISA kit (MyBioSource, Inc., San Diego, CA, USA). The inter- and intra-assay coefficients of variation were 12.3% and 9.9% for P4 and 10.2% and 5.7% for PRL, respectively.

### Statistical analysis

Statistical analysis was conducted using SAS (version 9.4 SAS Institute, USA). Data were evaluated using PROC LOGISTIC, PROC GLM, and PROC FREQ in SAS. In total, 2100 cows with complete hormonal protocols were included in the final statistical analysis. The model included treatment group effects (PG7G-63 vs. PG7G-73), parity (primiparous vs. multiparous), season (hot vs. moderate), and their interactions. To carry out the statistical analyses, data were coded as 1 (pregnant) or 0 (not pregnant) per AI on days 33 and 47 after TAI. The PROC GLM procedure was used to test independent variables (P4, PRL, and milk production) between treatment groups, parity, and season. A chi-square analysis was conducted using PROC FREQ to compare pregnancy per artificial insemination (P/AI) rates on days 33 and 47, as well as pregnancy loss rates between the treatment groups. The effects of the average weekly milk yield for the first 4 months and environmental data during the experimental period on the groups and pregnancy rates were estimated. Furthermore, the correlation between PRL concentration and average milk yield was evaluated at calving, GnRH, PGF_2α_, TAI, day 7 post-TAI, and day 7 and 14 post-TAI. The least squares mean for significant effects was compared at p < 0:05 and as a tendency at p < 0.10 using the t-test.

## RESULTS

The average milk production during the first 4-month pp did not differ (p = 0.30) between PG7G-63 and PG7G-73 treatment groups and averaged 28.0 ± 0.3 and 28.4 ± 0.3 Kg, respectively.

### Effect of TAI protocol and pregnancy on plasma P4 and PRL concentrations.

The proportions of cows with functional CL (plasma P4 [≥1 ng/mL]) at the first GnRH and PGF_2α_ injection were 80% and 90%, respectively. The effects of different hormonal treatment groups and pregnancy on P4 and PRL concentrations are shown in Tables 1 and 2. Plasma P4 and PRL concentrations did not differ between the two groups at the time of blood sampling. However, P4 levels were higher in pregnant cows than in non-pregnant cows at PGF_2α_ injection (p = 0.01), TAI, 7-day post-TAI (p = 0.05), and day 14 post-TAI (p = 0.01). Pregnant cows had a higher likelihood (p = 0.06) of being in the first GnRH injection group according to the Ovsynch protocol. Conversely, PRL levels were significantly lower in pregnant cows than in non-pregnant cows at PGF_2α_ injection (p = 0.01), TAI (p = 0.01), and day 7 post-TAI (p = 0.01) and showed a trend toward lower levels (p = 0.06) on day 14 post-TAI.

**Table 1:** Effect of TAI groups and pregnancy on mean (± SEM) plasma progesterone (P4) concentration (ng/mL) at calving, at first GnRH, PGF_2α_, TAI of Ovsynch protocol, and 7 and 14 days after AI.

Variable	Progesterone (P4) concentration ng/mL (± SEM)					

	At calving	At First GnRH	At PGF_2α_	At TAI	At 7-day post-TAI	At 14-day post-TAI
Groups^1^						
PG7G-63	0.9 ± 0.21	3.6 ± 1.04	6.2 ± 0.73	1.8 ± 0.24	6.3 ± 0.86	7.5 ± 0.79
PG7G-73	1.0 ± 0.18	4.5 ± 0.83	5.0 ± 0.58	2.2 ± 0.19	7.3 ± 0.69	7.0 ± 0.63
p-value	0.95	0.51	0.22	0.17	0.39	0.61
Pregnancy						
+	0.7 ± 0.18	5.4 ± 0.84	7.0 ± 0.70	0.7 ± 0.19	8.0 ± 0.70	8.9 ± 0.66
-	1.2 ± 0.21	2.7 ± 1.03	4.2 ± 0.61	3.3 ± 0.24	5.6 ± 0.85	5.6 ± 0.79
p-value	0.15	0.06	0.01	0.01	0.05	0.01

1 PG7G-63=Cows received PGF_2α_ injection on day 30 postpartum then another PGF_2α_ 14 days later, GnRH 2 days later, and a 7-day Ovsynch protocol (GnRH, 7 days, PGF_2α_ 56 h, GnRH, 16 h & TAI on day 63 postpartum). PG7G-73=Cows received PGF_2α_ injection on day 40 postpartum, then another PGF_2α_ 14 days later, GnRH 2 days later, and a 7-day Ovsynch protocol (GnRH, 7 days, PGF_2α_ 56 h, GnRH, 16 h & TAI on day 73 postpartum). TAI=Timed artificial insemination, SEM=Standard error of the mean

**Table 2:** Effect of TAI groups and pregnancy on mean (± SEM) plasma PRL concentration (ng/mL) at calving, at the first GnRH, PGF_2α_, TAI of Ovsynch, and 7 and 14 days after AI.

Variable	PRL concentration ng/ml (± SEM)					

	At calving	At First GnRH	At PGF_2α_	At TAI	At 7-day post-TAI	At 14-day post-TAI
Groups^1^						
PG7G-63	137 ± 16	95 ± 5	85 ± 3	77 ± 4	71 ± 4	63 ± 5
PG7G-73	152 ± 15	104 ± 5	93 ± 3	83 ± 3	79 ± 4	71 ± 5
p-value	0.49	0.19	0.10	0.19	0.17	0.23
Pregnancy						
+	136 ± 15	94 ± 5	82 ± 3	72 ± 3	67 ± 4	60 ± 5
-	153 ± 17	105 ± 5	96 ± 4	88 ± 4	83 ± 4	74 ± 5
p-value	0.48	0.19	0.01	0.01	0.01	0.06

1 PG7G-63=Cows received PGF_2α_ injection on day 30 postpartum then another PGF_2α_ 14 days later, GnRH 2 days later, and a 7-day Ovsynch protocol (GnRH, 7 days, PGF_2α_ 56 h, GnRH, 16 h & TAI on day 63 postpartum). PG7G-73=Cows received PGF_2α_ injection on day 40 postpartum then another PGF_2α_ 14 days later, GnRH 2 days later, and a 7-day Ovsynch protocol (GnRH, 7 days, PGF_2α_ 56 h, GnRH, 16 h & TAI on day 73 postpartum). TAI=Timed artificial insemination, SEM=Standard error of the mean, PRL=Prolactin

Milk production was significantly lower (p = 0.05) in pregnant cows than in non-pregnant cows at all time points: PGF_2α_ injection (24.5 ± 2 vs. 34.7 ± 2 kg/day), TAI (24.2 ± 2 vs. 35.2 ± 2 kg/day), day 7 post-TAI (24.6 ± 2 vs. 34.4 ± 2 kg/day), and day 14 post-TAI (25.2 ± 2 vs. 33.9 ± 2 kg/day). Table 3 presents the Pearson correlation coefficients between PRL levels and average milk production at calving and throughout the PG7G protocol. A positive correlation was observed between PRL and milk yield at calving. No correlations were detected between these variables on other sampling days. A positive relationship was evident between PRL concentrations and milk production at the time points of GnRH, PGF_2α_, TAI, and day 7 and day 14 post-TAI.

**Table 3:** Pearson correlation coefficients between plasma PRL concentration and mean milk yield at calving, first GnRH, PGF_2α_, TAI, and 7 and 14 days after AI.

Milk yield at	PRL on (day)					

	Calving	GnRH	PGF_2α_	TAI	7d post-AI	14d post-AI
Calving	0.54**	0.32^ns^	0.16^ns^	0.20^ns^	0.14^ns^	0.10^ns^
GnRH	0.52**	0.54**	0.69**	0.74**	0.73**	0.73**
PGF_2α_	0.48*	0.52**	0.70**	0.75**	0.76**	0.76**
TAI	0.47*	0.51*	0.67**	0.75**	0.73**	0.71**
7d post-AI	0.49*	0.57**	0.71**	0.76**	0.76**	0.75**
14d post-AI	0.53**	0.57**	0.71**	0.74**	0.76**	0.75**

** p < 0.01,

* p < 0.05. TAI=Timed artificial insemination, PRL=Prolactin

### Effect of treatment groups, parity, and season on pregnancies per AI (P/AI)

Logistic regression analysis (Table 4) showed no significant association between P/AI on Day 33 in the treatment groups (PG7G-63: 40.2% vs. PG7G-73: 42.7%, p = 0.447), parity (Primiparous: 41.0% vs. Multiparous: 40.9%, p = 0.332), or season (Hot: 43.2% vs. Moderate: 39.8%; p = 0.536). In contrast, P/AI on day 47 was significantly influenced by treatment group (PG7G-63: 31.7% vs. PG7G-73: 37.9%, p = 0.034) and season (Hot: 32.4% vs. Moderate: 36.2%, p = 0.032) but not by parity (Primiparous: 37.0% vs. Multiparous: 33.6% (p = 0.238) (Table 5). PLs between days 33 and 47 after TAI are presented in Table 6. Logistic regression revealed that PLs were significantly associated with treatment groups (PG7G-63: 21.2% vs. PG7G-73: 9.0%, p = 0.001), parity (primiparous: 9.7% vs. multiparous: 17.9%, p = 0.001), and season (hot: 25.0% vs. moderate: 9.1%, p = 0.001).

**Table 4:** PR at 33 days after TAI in different hormonal treatment groups, cow parity, and season, and the combined effects of these factors.

Variable	Class	Pregnancies per AI % (n)	OR^1^	p-value
Group^2^	PG7G-63	40.2 (416/1034)	R	0.447
	PG7G-73	41.7 (444/1066)	1.055	
Parity	Primiparous	41 (320/781)	R	0.332
	Multiparous	40.9 (540/1319)	1.002	
Season	Hot	43.2 (312/723)	R	0.536
	Moderate	39.8 (548/1377)	1.146	
Group × Parity	PG7G-63 × Prim	41.9 (172/411)	R	0.308
	PG7G-63 × Mult	39.2 (244/623)	1.053	
	PG7G-73 × Prim	40 (148/370)		
	PG7G-73 × Mult	42.5 (296/696)		
Parity × Season	Prim × Hot	37.1^c^ (108/291)	R	0.001
	Prim × Moderate	43.3^b^ (212/490)	0.874	
	Mult × Hot	47.2^a^ (204/432)		
	Mult × Moderate	37.9^c^ (336/887)		

1 OR=Odds ratio (95% CI); R=OR reference.

2 PG7G-63: Cows received PGF_2α_ injection on day 30 postpartum then another PGF_2α_ 14 days later, GnRH 2 days later and a 7-day Ovsynch protocol (GnRH, 7 days, PGF_2α_ 56 h, GnRH, 16 h & TAI on day 63 postpartum). PG7G-73: Cows received PGF_2α_ injection on day 40 postpartum then another PGF_2α_ 14 days later, GnRH 2 days later and a 7-day Ovsynch protocol (GnRH, 7 days, PGF_2α_ 56 h, GnRH, 16 h & TAI on day 73 postpartum). ^a,b,c^The percentages among treatments and parity with different superscripts differ (p < 0.05). PR=Pregnancy rate, TAI=Timed artificial insemination

**Table 5:** PR at 47 days after TAI in different hormonal treatment groups, cow parity, and season, and the combined effects of these factors.

Variable	Class	Pregnancies per AI % (n)	OR^1^	p-value
Group^2^	PG7G-63	31.7^b^ (328/1034)	R	0.034
	PG7G-73	37.9^a^ (404/1066)	0.748	
Parity	Primiparous	37.0 (289/781)	R	
	Multiparous	33.6 (443/1319)	1.192	
Season	Hot	32.4^b^ (234/723)	R	0.032
	Moderate	36.2^a^ (498/1377)	1.211	
Group × Parity	PG7G-63 × Prim	37^a^ (152/411)	R	
	PG7G-63 × Mult	28.3^b^ (176/623)	0.628	
	PG7G-73 × Prim	37^a^ (137/370)		
	PG7G-73 × Mult	37.4^a^ (267/696)		
Parity × Season	Prim × Hot	30.9^b^ (90/291)	R	0.035
	Prim × Moderate	40.6^a^ (199/490)	0.984	
	Mult × Hot	33.3^b^ (144/432)		
	Mult × Moderate	33.7^b^ (299/887)		

1 OR=Odds ratio (95% CI); R=OR reference.

2 PG7G-63: Cows received PGF_2α_ injection on day 30 postpartum then another PGF_2α_ 14 days later, GnRH 2 days later and a 7-day Ovsynch protocol (GnRH, 7 days, PGF_2α_ 56 h, GnRH, 16 h & TAI on day 63 postpartum). PG7G-73: Cows received PGF_2α_ injection on day 40 postpartum then another PGF_2α_ 14 days later, GnRH 2 days later and a 7-day Ovsynch protocol (GnRH, 7 days, PGF_2α_ 56 h, GnRH, 16 h & TAI on day 73 postpartum). ^a,b^The percentages of treatments and parity with different superscripts differed (p < 0.05). PR=Pregnancy rate, TAI=Timed artificial insemination

**Table 6:** Pregnancy losses between days 33 and 47 after TAI in different hormonal treatment groups, cow parity, and season, and the combined effects of these factors.

Variable	Class	Pregnancy losses % (n)	OR^1^	p-value
Group^2^	PG7G-63	21.2^a^ (88/416)	R	0.001
	PG7G-73	9.0^b^ (40/444)	3.145	
Parity	Primiparous	9.7^b^ (31/320)	R	0.003
	Multiparous	17.9^a^ (97/540)	0.455	
Season	Hot	25.0^a^ (78/312)	R	0.001
	Moderate	9.1^b^ (50/548)	0.285	
Group × Parity	PG7G-63 × Prim	11.6^b^ (20/172)	R	0.171
	PG7G-63 × Mult.	28.9^a^ (68/244)	6.912	
	PG7G-73 × Prim	7.4^b^ (11/148)		
	PG7G-73 × Mult	9.8^b^ (29/296)		
Group × Season	PG7G-63 × Hot	28.9^a^ (42/145)	R	0.054
	PG7G-63 × Moderate	16.9^b^ (46/271)	11.035	
	PG7G-73 × Hot	21.6^a^ (36/167)		
	PG7G-73 × Moderate	1.4^c^ (4/277)		

1 OR=Odds ratio (95% CI), R=OR reference.

2 PG7G-63: Cows received PGF_2α_ injection on day 30 postpartum then another PGF_2α_ 14 days later, GnRH 2 days later and a 7-day Ovsynch protocol (GnRH, 7 days, PGF_2α_ 56 h, GnRH, 16 h & TAI on day 63 postpartum). PG7G-73: Cows received PGF_2α_ injection on day 40 postpartum then another PGF_2α_ 14 days later, GnRH 2 days later and a 7-day Ovsynch protocol (GnRH, 7 days, PGF_2α_ 56 h, GnRH, 16 h & TAI on day 73 postpartum). ^a,b,c^Percentage differences among treatments and parity with different superscripts differ (p < 0.05). TAI=Timed artificial insemination

### Interaction effect of treatment groups, parity, and season on pregnancies per AI (P/AI)

No interaction was observed between treatment groups and either parity or season at 33 days post-TAI (p = 0.308 and p = 0.547, respectively). However, a significant interaction was found between parity and season (p = 0.001). Multiparous cows exhibited higher P/AI in the hot months (47.2%) than in moderate (37.9%), whereas primiparous cows displayed higher P/AI in the moderate compared to hot (43.3% vs. 37.1%, respectively, Table 4). Conversely, significant interactions were detected between treatment groups and parity (p = 0.033) and between parity and season p = 0.035, Table 5) at 47 days after TAI but not between treatment groups and season (p = 0.232). Primiparous cows in the PG7G-63 group exhibited higher P/AI rates (37%) than multiparous cows in the same group (28.3%), whereas no difference in P/AI was observed between primiparous and multiparous cows in the PG7G-73 group (37% vs. 37.4%, respectively). In addition, primiparous cows had higher P/AI under moderate conditions (40.6%) than under hot conditions (30.9%), whereas no difference in P/AI was found between multiparous cows under hot (33.3%) and moderate (33.7%) conditions.

No interaction was detected between treatment groups and parity or between parity and season on PLs (p = 0.171 and p = 0.679, respectively, Table 6). However, an interaction was found between treatment groups and season (p = 0.054). The cows experienced higher PL rates in the hot season (28.9%) than in the moderate season (16.9%). Moreover, the cows in the PG7G-73 group exhibited increased PLs in the hot season (21.6%) relative to the moderate season (1.4%).

**Figure 2:**
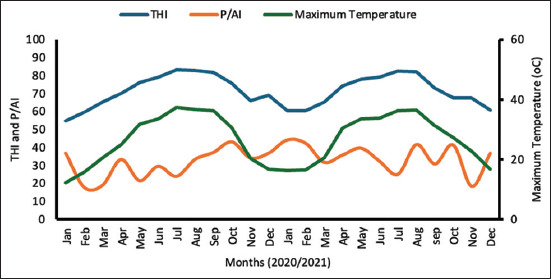
PR, P/AI, THI, and maximum temperature throughout the study period. PR=Pregnancy rate, P/AI=Per artificial insemination, THI=Temperature–humidity index.

**Figure 3:**
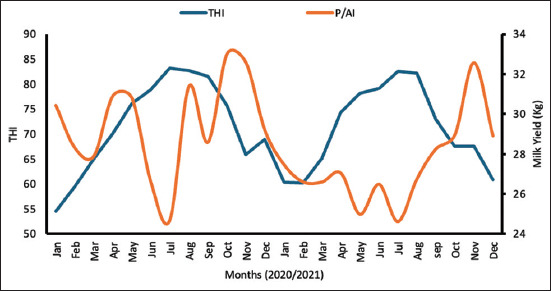
Milk yield (Kg) with THI throughout the study period. P/AI=Per artificial insemination, THI=Temperature–humidity index.

The average THI was significantly higher (p = 0.001) in the hot season (81.3) than in the moderate season (66.8). A substantial 76% of the days in the hot period experienced THI values exceeding the 72 threshold, indicating significant heat stress for the cows. Figure 2 illustrates the relationship between maximum temperature, THI, and P/AI, independent of the treatment group. P/AI was significantly lower (p = 0.05) during the hot months of June to September (THI > 72) compared with the remaining study period. In addition, average milk production was markedly reduced (p = 0.001) in the hot season (27.5 ± 0.4 kg) compared with the moderate season (28.9 ± 0.3 kg) (Figure 3).

## DISCUSSION

To the best of our knowledge, this is the first study to investigate the effects of extending the VWP from 63 to 73 DIM following the PG7G synchronization protocol on progesterone (P4), PRL, P/AI, PL, and parity in lactating Holstein cows under summer and winter conditions. The proportion of cows with detectable progesterone concentrations during the Ovsynch phase in both PG7G groups aligned with previous research by Dirandeh *et al*. [21]; they observed that a higher percentage of cows in the PG7G group had plasma progesterone levels of ≥ 1 ng/ml at the first GnRH (81.6%), at the PGF_2α_ of Ovsynch (85.0%), and at the second GnRH treatment (78.3%). Heidari *et al*. [32] investigated a modified G6G protocol and found that extending the interval between PGF_2α_ and GnRH during presynchronization increased the percentage of cows with detectable progesterone levels at the first GnRH and PGF_2α_ injections. Although their study focused on a different protocol, the results underscore the importance of ovarian status in successful synchronization programs.

The plasma P4 concentration was lower in both groups at parturition, consistent with previous findings by Gross *et al*. [37]. Pp progesterone levels gradually increased, with mean values of approximately 2 ng/mL at TAI, rising to 3–4 ng/mL by day 14 post-TAI. These results are consistent with those reported by Vukovic *et al*. [38]. However, individual variability was observed, as evidenced by the wide range of progesterone concentrations at TAI (0.0–9.94 ng/mL) reported by Colazo *et al*. [39]. A particularly interesting study by Cummins *et al*. [40] reported that one of the major hormonal differences found in cows with genetics for high fertility was 34% greater circulating P4 concentrations than in cows with poor genetic merit for fertility. While Santos *et al*. [23] reported lower progesterone concentrations in a Double-Ovsynch protocol, the discrepancies might be attributed to differences in study design or population characteristics.

Consistent with the findings of Vukovic *et al*. [38], plasma progesterone (P4) concentrations were lower at the time of artificial insemination (AI) but elevated at 7 and 14-day post-AI in pregnant cows compared with non-pregnant cows. This differential P4 profile between pregnant and non-pregnant animals after AI or embryo transfer has been observed in multiple studies [39, 41]. More recently, Madureira *et al*. [42] reported that cows with lower P4 concentrations at 7, 14, and 21-day post-spontaneous estrus exhibited decreased pregnancy rates following AI, suggesting a potential link between early-stage P4 levels and fertility outcomes.

Plasma PRL levels peaked at parturition (145 ng/mL) and then gradually declined, stabilizing around 77–88 DIM. A positive correlation was observed between PRL and milk production. Pregnant cows exhibited lower PRL levels than non-pregnant cows. These findings are consistent with those of previous studies by Edgerton and Hafs [8], demonstrating a prepartum PRL surge followed by a decline during early lactation. The impact of PRL on milk production in dairy cows is complex and context-dependent. For instance, administering recombinant PRL for 14 days during early or late lactation had no significant effect on milk yield [43]. However, studies have shown that cows respond to exogenous PRL during early lactation [44]. Although PRL has been implicated in stimulating milk production [45], its effect is influenced by various factors, including the lactation stage and hormonal concentration [46]. The mammary gland’s responsiveness to PRL appears to be dynamic, varying across different lactation phases [47].

The overall pregnancy rates in this study were 40.9% on day 33 and 34.9% on day 47, resulting in a 14.9% PL. These findings align with Dirandeh *et al*. [21], who reported pregnancy rates of approximately 44% and 39% on days 32 and 60, respectively, following a modified Ovsynch protocol. However, pregnancy rates were lower in the current study. Potential factors contributing to this discrepancy include seasonal variations in temperature between summer and winter and the inclusion of both primiparous and multiparous cows. Furthermore, Shahzad *et al*. [22] assessed three ovulation synchronization protocols in lactating dairy cows and found that the PG7G protocol had a pregnancy rate of 56% at 30 days and 53% at 60 days, although direct comparisons are limited due to differing study populations and methodologies.

On the other hand, the P/AI on day 47 was influenced by multiple factors, including treatment group, season, and parity. PL was affected by similar factors, with the added influence of the interaction between the treatment group and the season. In Jordan, most farms use VWPs of approximately 50–60 DIM to inseminate their cows after calving. Previous studies by Alnimer *et al*. [19] and Alnimer and Ababneh [48] at the same farm combined AI for detecting estrus and TAI programs at the same VWP durations and found that P/AI at first insemination was approximately 31% and 33%. The current study extended the VWP to 73 days using the PG7G protocol and achieved a higher pregnancy rate in the PG7G-73 group than in the PG7G-63 group. In other synchronization protocols, various studies evaluated that the pregnancy rates of cows following the implementation of the G6G or G7G protocols have been reported in the range of 26.8%–54% inseminated at VWP between 50 and 82 DIM [13, 30–32]. For instance, lower pregnancy rates were reported with a modified G7G protocol at 73 DIM, but higher rates were reported with intermediate heat detection [15]. In addition, the pregnancy rates of cows following the implementation of the Presynch-Ovsynch, Presynch-Ovsynch + CIDR, or Double Ovsynch protocols have been reported in the range of 28.4%–47% between 42 and 88 DIM [28, 49]. Therefore, the observed increase in pregnancy rates with extended VWP is in agreement with previous research by Stangaferro *et al*. [50] and Gobikrushanth *et al*. [51]. However, direct comparisons between studies are limited because of variations in experimental protocols, study populations, and environmental conditions.

Regardless of treatment groups, the overall first service P/AI was similar between primiparous and multiparous cows, with an interaction between treatment groups and parity or parity and season. Conversely, PLs were higher in multiparous cows, particularly when considering interactions between treatment groups and seasons. Previous studies by Astiz and Fargas [30] and El-Tarabany [49] have also reported similar P/AI rates between primiparous and multiparous cows. However, another study by Yousuf *et al*. [31] reported that primiparous cows tend to have higher pregnancy rates than multiparous cows when using G6G or modified G7G protocols. Astiz and Fargas [30] suggested a potential advantage of using the G6G protocol in multiparous cows at later insemination times. The primiparous cows in the PG7G-63 group exhibited higher pregnancy rates than the multiparous cows in the same group, whereas no difference was observed between the parities in the PG7G-73 group. This finding aligns with Abdel Aziz and Abdel-Wahab [18], who reported higher pregnancy rates for primiparous cows using a 12-day Presynch–Ovsynch protocol when inseminated before 70-day postpartum. Conversely, a trend toward higher pregnancy rates was observed in multiparous cows that inseminated between 70 and 80-day postpartum. Contrastingly, Stangaferro *et al*. [50] found that cows following the Double Ovsynch protocols achieved higher pregnancy rates to the first AI in primiparous than in multiparous cows inseminated at 60 and 88-day pp. While primiparous cows in the current study’s PG7G-63 group demonstrated higher pregnancy rates, potential explanations include reduced metabolic challenges [52] and improved luteolysis compared with multiparous cows [53] and different endocrine responses [54]. In addition, the lower P/AI in the PG7G-63 multiparous cows might be attributed to an increased risk of PL. It is well documented that P/AI decreases with increasing lactation [55, 56].

In this study, the treatment groups were associated with P/AI, but heat stress was identified as the primary influencing factor. The overall P/AI on day 47 was higher in moderate than in hot months. In addition, when the temperature-humidity index (THI) exceeded 72, P/AI was lower than that recorded in the remaining months of the experimental period. These findings are in line with previous research by Alnimer *et al*. [27], Nanas *et al*. [57], and Changtes *et al*. [58], reporting higher P/AI in dairy cows during the winter season than during the summer months.

As highlighted by Djelailia *et al*. [59], Holstein dairy cows raised in hot, arid environments are highly susceptible to heat stress a few days before, during, or shortly after breeding. This heightened susceptibility, particularly when the THI exceeds 70, can significantly impact reproductive performance, including decreased pregnancy rates per AI. This finding is consistent with studies by Renaudeau *et al*. [60], Giannone *et al*. [61], and Das *et al*. [62], which have demonstrated a negative correlation between elevated THI and fertility metrics in dairy cattle. The harmful effects of heat stress on reproductive function are multifaceted and include disruption of hormonal profiles, impaired oocyte quality, altered uterine environment, and reduced libido. Furthermore, Schüller *et al*. [63] suggested that the pregnancy rate steadily decreases as the THI value rises from 51 to 73, with the lowest conception rate observed at THI ≥72 on the day of estrus. High temperatures adversely affect fertility through various mechanisms, including impaired steroidogenesis, compromised oocyte quality, and reduced progesterone production by the corpus luteum (CL), as well as the fertilization rate [64, 65]. Furthermore, heat stress disrupts oocyte maturation, fertilization, and early embryo development. Researchers have also noted that during the warm season, there is an imbalance in the hypothalamic-pituitary-ovarian axis, leading to reduced reproductive performance and compromised oocyte quality in cows [20, 65–67].

Primiparous cows exhibited higher pregnancy rates during moderate periods than during hot periods, whereas pregnancy rates remained consistent across seasons for multiparous cows. These findings align with those of Astiz and Fargas [30], who reported reduced fertility in primiparous cows during hot seasons. Conventionally, multiparous cows are considered more susceptible to heat stress [68, 69]. However, recent research suggests that cows with more lactation may better adapt to hot environments and exhibit reduced heat stress behaviors compared to primiparous cows [70].

The occurrence of PL was significantly influenced by the treatment group, parity, season, and the interaction between groups and seasons. The multiparous cows experienced greater PLs than the primiparous cows, which is consistent with the findings of previous studies by Abdel Aziz and Abdel-Wahab [19], Santos *et al*. [26], and Alnimer *et al*. [27]. In addition, a previous study by Lean *et al*. [71] demonstrated that the frequency of embryonic mortality increases from the 1^st^ to the 3^rd^ parity. However, Chebel *et al*. [72] have reported that parity had no effect on PL. Furthermore, higher ambient temperatures from June to September (hot season) resulted in a significantly higher incidence of PLs compared with the other months of the year. Similarly, Drost *et al*. [73] and Cartmill *et al*. [74] observed PL rates of 65% and 42.7%, respectively, in high-producing cows exposed to heat stress. Our previous research by Alnimer *et al*. [19] also found higher PL rates during summer than during cooler periods. Nanas *et al*. [57] reported increased early embryonic death rates during summer than during winter. The variability in PL rates across studies may be attributed to differences in geographic location, study population, and specific environmental conditions, emphasizing the complex relationship between heat stress and reproductive performance in dairy cows.

Our results are consistent with prior research, demonstrating that high summer temperatures (THI > 72) adversely affect milk production [75–77]. Consistent with our observations, Stojnov *et al*. [78] reported lower milk yields in cows calving during the summer months, highlighting the harmful effects of heat stress on lactation performance.

## CONCLUSION

The study demonstrates that extending the VWP to 73-day pp improves pregnancy rates in lactating Holstein cows, particularly during moderate climatic conditions, and highlights the interplay of hormonal treatments, parity, and seasonal variations in reproductive outcomes. However, the study is limited by its reliance on a single farm, which may constrain the generalizability of results and the exclusion of environmental factors beyond temperature and humidity that could affect fertility. Future research should investigate broader environmental influences, refine synchronization protocols for diverse farm systems, and explore the mechanisms underlying the relationship between prolactin levels, milk production, and reproductive success to optimize fertility and farm profitability across varying conditions.

## AUTHORS’ CONTRIBUTIONS

MAA: Designed the experiment, developed the methodology, drafted the manuscript, and conducted the statistical analysis. MAAM, MAQ, and AIS: Performed the blood sample analysis. MAA, MAAM, MAQ, and AIS: Conducted study and data collection. MAAM: Literature search. All authors have read and approved the final manuscript.
